# Predictable Irreversible Switching Between Acute and Chronic Inflammation

**DOI:** 10.3389/fimmu.2018.01596

**Published:** 2018-08-07

**Authors:** Abulikemu Abudukelimu, Matteo Barberis, Frank A. Redegeld, Nilgun Sahin, Hans V. Westerhoff

**Affiliations:** ^1^Department of Synthetic Systems Biology and Nuclear Organization, Swammerdam Institute for Life Sciences, University of Amsterdam, Amsterdam, Netherlands; ^2^Department of Molecular Cell Physiology, VU University Amsterdam, Amsterdam, Netherlands; ^3^Division of Pharmacology, Department of Pharmaceutical Sciences, Faculty of Science, Utrecht University, Utrecht, Netherlands; ^4^School for Chemical Engineering and Analytical Science, The Mill, University of Manchester, Manchester, United Kingdom

**Keywords:** inflammation, innate immunity, cross-reacting antigen, modeling, irreversible transition, bi-stability

## Abstract

Many a disease associates with inflammation. Upon binding of antigen-antibody complexes to immunoglobulin-like receptors, mast cells release tumor necrosis factor-α and proteases, causing fibroblasts to release endogenous antigens that may be cross reactive with exogenous antigens. We made a predictive dynamic map of the corresponding extracellular network. *In silico*, this map cleared bacterial infections, *via* acute inflammation, but could also cause chronic inflammation. In the calculations, limited inflammation flipped to strong inflammation when cross-reacting antigen exceeded an “On threshold.” Subsequent reduction of the antigen load to below this “On threshold” did not remove the strong inflammation phenotype unless the antigen load dropped below a much lower and subtler “Off” threshold. In between both thresholds, the network appeared caught either in a “low” or a “high” inflammatory state. This was not simply a matter of bi-stability, however, the transition to the “high” state was temporarily revertible but ultimately irreversible: removing antigen after high exposure reduced the inflammatory phenotype back to “low” levels but if then the antigen dosage was increased only a little, the high inflammation state was already re-attained. This property may explain why the high inflammation state is indeed “chronic,” whereas only the naive low-inflammation state is “acute.” The model demonstrates that therapies of chronic inflammation such as with anti-IgLC should require fibroblast implantation (or corresponding stem cell activation) for permanence in order to redress the irreversible transition.

## Introduction

An inflammatory environment engages a network of innate and adaptive immune cells (1–4), tissue components like stromal fibroblasts (5), extracellular matrix (6), the vascular networks of blood and lymphatics (7), and soluble molecular messengers like plasma proteins, cytokines, and chemokines (8). The inflammatory process has been classified into acute and chronic substantiations. Once the body has been infected by pathogens, innate immune cells such as macrophages and mast cells express pattern recognition receptors (PRRs) that may become ligated by pathogen-associated molecular patterns (PAMPs). PRR activation leads to activation of innate immune cells and pro-inflammatory immune responses against the pathogen. In addition, PRR activation of antigen-presenting cells, like dendritic cells (DCs), eventually induces adaptive immune responses. Next to PAMPs, specific pathogen-independent structures deriving from damaged or dying cells [“damage-associated molecular patterns (DAMPs)” (9)] activate. They may signal a status of altered-self. DAMPs activate innate cells like DCs and macrophages *via* specific PRRs, i.e., toll-like receptors and C-type lectin receptors. DAMPs may thus amplify immunity against pathogens, but also promote autoimmunity in sterile ongoing inflammatory processes.

Acute inflammation facilitates innate and acquired immunity, which combats infection by attracting leukocytes and plasma proteins (and at a later stage antibodies) to sites of infection or injury (10, 11). Prolonged or more intensive infiltration by various immune cells may turn acute inflammation into chronic inflammation (12, 13). Acute inflammations persist for a couple of days or weeks; they require the presence of the external stimulus. Prolonged or more intensive infiltration by various immune cells may turn acute inflammation into chronic inflammation, which persists over months or years, well beyond the presence of the external stimuli. DAMPs may be involved in both stages of inflammation.

In some autoimmune diseases such as inflammatory arthritis (14, 15) and multiple sclerosis (16, 17), chronic inflammation may be caused by some interplay between B-cell activation and differentiation, autoantibody formation, and mast-cell function. Antigen binds to a specific B cell receptor (BCR), which consists of a monomeric membrane-bound immunoglobulin (Ig), composed of two light chains and two heavy chains (18). In addition to intracellular BCR signaling *via* the Ig-associated Igα and Igβ, the activated B cell requires signals to survive, proliferate, and eventually differentiate into memory B cells and Ig secreting plasma blasts and plasma cells (19). For thymus-independent antigens, the second signal may consist of PRR ligation (20, 21). Thymus-dependent antigens require CD40 co-stimulation and cytokines from cognate CD4 T cells (22, 23), such as the follicular T helper (Tfh) cells (24, 25). Tfh cells mediate B cell class switching and somatic hypermutation, yielding high-affinity IgG and IgE (26, 27). Igs infiltrating a site of infection bind the specific pathogen or autoantigen. This supports pathogen eradication. Both pathogen-specific Igs and auto-antigen-specific Igs may then also instigate inflammation.

Usually, inflammation is initiated by innate immunity against exogenous antigens; either be allergens (such as pollen) or parts of microorganisms (such as coat or toxin). Exogenous antigens can be cross-reactive with endogenous antigens: *Streptococcus mutans* antigen is highly cross-reactive with heart tissue antigen (28). Antigenic cross-reactivity was detected by using cartographic comparison between H5N1 influenza virus and other virus strains (29). Potential cross-reactivity of all MHC class I cancer immunotherapy antigens assessed by quantifying cross-reactivity for MHC-1 epitopes was distributed across tissues (30). Every inflammatory disease appears linked to one or more infectious agents. Infectious antigens may impact the autoimmune response by *molecular mimicry* (31). B-cells that are activated in response to the antigens are then also cross-reactive to self and may lead to further activation of T cells and mast cells.

B cell differentiation triggered by contact with antigen and with cytokines produced by cognate Tfh cells induces secretion of antibodies (here referred to as Ig’s). Two types of immunoglobulin light chains (IgLC) are produced in human: kappa (κ) and lambda (λ) type. Although B-cell activation causes production of excess immunoglobulin light chains that are not bound to heavy chains (FLCs) (32), we shall here focus on their complex, i.e., IgE: in healthy individuals, the majority of light chains in serum are bound to heavy chains. IgLC can serve as a drug target (33), with the F991 peptide as drug example. Nakano et al. reviewed that this peptide may also bind to the CDR3 region of the FLC which remains exposed to the medium when the light chain is in its complex with heavy chain (34).

Mast cells are effector cells of the innate immune system. Capable of producing proteolytic enzymes, cytokines, and growth factors, they are innate to many normal tissues and organs. The tissue environment matures these mast cells through cytokines such as stem cell factor (SCF) and nerve growth factor (35), which acts *via* tyrosine kinase receptors (TrkA, B, and C) different from C-kit activated SCF (36). The mast cell is primarily activated by IgE, but molecules such as SCF, IgG, FLC, hormones, complement peptides (C3a, C4a, and C5a), and certain neuropeptides also trigger mast cell activation. The activated mast cells secrete pro-inflammatory mediators such as tumor necrosis factor-α (TNF-α) (37), tryptase (38), and vascular endothelial growth factor (VEGF) (39).

Antigen-dependent mast cell activation is mainly mediated by IgE through cross-linking of their surface receptor FcεRI (40) at a binding affinity of 10^9^–10^10^ M^−1^ (41). FcεRI has seven N-linked glycosylation sites in its 176 amino acid residues. The intact receptor on mast cells is approximately 40% carbohydrate by its ~50 kDa mass (42, 43). The Fc fragment of IgE interacts with the extracellular domains of FcεRI-α chain (40, 44). IgE levels are high in serum and synovial fluid of patients with rheumatoid arthritis (45) or allergies (46).

The interplay of these many factors in the inflammatory network correlates with the research findings that innate immune cells and their mediators promote inflammation (47, 48). Although it operates largely extracellularly, this network has a number of aspects in common with the various intracellular signal transduction networks that have increasingly become subject to systems biology studies: it is complex, co-determined by the individual’s genome, affected by environmental factors and, it exhibits memory effects, such as related to prior exposure to external antigens leading to enhanced reactivity (49–51). Diseases related to some of these features or their absences have been recognized as systems biology diseases (52).

In this paper, we therefore build and then examine a computational systems biology model of IgE-induced inflammation. We focus on IgE-mediated mast-cell activation and a relatively small number of factors and interactions, so that the model’s results can also be understood more intuitively. The resulting model makes existing knowledge about the components of the network and their interactions, predictive of the response to various antigen doses. The predictions include that the network can flip from weak to strong inflammation, but not back. A peptide drug interfering with IgLC may transiently reset the network from a chronic inflammation state to an uninflamed state, but this cure may not persist. Perhaps surprisingly, naive fibroblasts may play a decisive role in the final outcome of such a therapy.

## Materials and Methods

**Figure 1:**
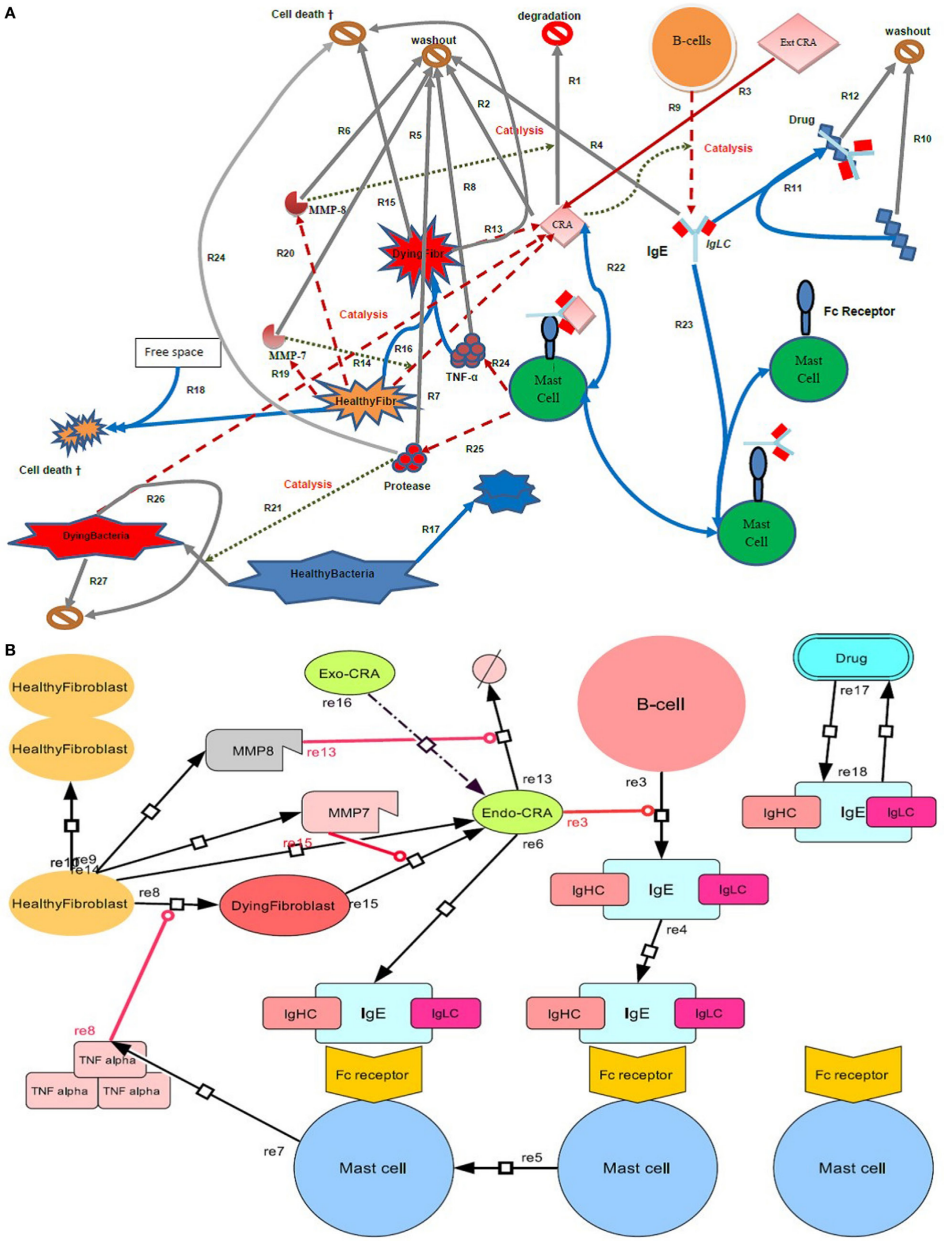
Diagram of the inflammatory network studied here. **(A)** Four types of cell (B cells, mast cells, bacteria, and fibroblasts) are shown, where the latter are either in a healthy or a dying state. In terms of extracellular molecules, we consider Cross Reactive Antigen (CRA), IgE, TNF-α, the proteases MMP-7 and MMP-8, and drug molecules. The symbol 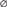 or 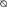 represents an exit into which all molecules wash out at a certain rate and cells disappear at a certain death rate. Free space refers to space not taken up by fibroblasts. Solid arrows refer to conversion processes, dashed arrows to secretion processes, and dotted arrows to catalysis or regulation. Processes are numbered as *R* followed by a number. **(B)** Systems Biology Graphical Notation (SBGN) scheme. By using the program Cell Designer, we designed an IgE-mediated inflammatory network, which corresponds with the network model as shown in **(A)**.

The native immune network as described in Figure 1 was decomposed into its component processes and for each of the latter, a characteristic rate equation was developed. This equation formulated the rate at which the process should proceed as a function of the concentrations of the components involved in the process, and kinetic parameters. For each species in Figure 1, a balance equation was then formulated, specifying its time dependence as the difference between the rates of the reactions synthesizing the species and the rates of the processes degrading it. Combination of the rate and balance equations then led to differential equations, which were integrated as functions of time. This computation process was performed using COPASI (a biochemical systems simulator). The dynamic model we made thereby depends not only on the topology of the network of Figure 1 but also on parameter values, to which we therefore had to assign values. For most of the parameters of our model, accurate values are unknown. We chose parameter values that were in a realistic range based on biochemical and physicochemical data and considerations, as specified below.

### Cell Concentrations, Total Space, Growth *k*_17_, *k*_18_

A fibroblast has a volume of some 2.0 pl (i.e., 10^−12 ^l) (53), giving a space completely packed with fibroblasts, a fibroblast concentration of almost 1.0 pM. We set the fixed concentration of B cells to 1 fM (i.e., 10^−15 ^mol/l) and the constant total concentration of the mast cells to 0.1 fM. We assumed that in the remaining space, 1,000 fM of healthy and dying fibroblasts could exist. We expressed this “total_space” in terms of the concentration of subspaces each of the volume of a fibroblast (2.0 pl). In this way, the total space was 1,000 fM and the concentration of healthy fibroblasts was 1,000 fM or less. Free_space was assumed to equal total_space minus the concentration of fibroblasts and expressed in unit fibroblast, i.e., in fM. The fibroblast division rate (*R*_18_) was made proportional to free space at a second order rate constant of *k*_18_ = 0.485/nM/min (nM refers to nanoMolar, i.e., 10^−9 ^mol/l; min refers to minute, equal to 60 s). When all space was free (and hence free_space = 1,000 fM), this corresponded to a 24 h doubling time. If present, bacteria were taken to grow (*R*_17_) at a specific growth rate of *k*_17_ = 0.01/min, at a rate independent of the free space, as bacteria were assumed to fit in between the larger mammalian cells.

### Association Rate Constants *k*_1_, *k*_9_, *k*_14_, *k*_16_, *k*_21_, *k*_22_, and *k*_23_

In water, the maximum diffusion-limited rate constant for association follows the Smoluchowski equation *k*_on_ = 4π*Dr* (54). Here, *r* is the sum of the radii of the two colliders and *D* their diffusion constant. This corresponds to 6 × 10^−5^/fM/min for small molecules and some 10^−5^/fM/min = 10.0/nM/min for proteins. When the two diffusing objects attract each other (or tend to remain together as in surface diffusion), the number can readily become 10 times higher (55). When the target is any of multiple receptors on a target cell, the association rate constant increases proportionally to the number of receptors per cell, but this only up to a maximum of some 5,000 per cell (56). When there are approximately 10^5^ receptors expressed on the surface of the cell (57), any collision with the cell will be effective and then the rate constant is increased by the factor of π·*r*_cell_/*r*_IgE_ ≈ 30,000. Combining all these effects and allowing by a factor of 2 for a reduction in diffusion rate due to steric hindrances, we obtained a diffusion-limited rate constant for IgE binding to IgE receptor-FcεRI (*R*_23_) on the mast cell of approximately 10/fM/min. In the next process (*R*_22_), a cross-reacting antigen (CRA) molecule associates with an IgE presented by a mast cell. This may happen when that mast cell contains only one IgE, but will happen faster when the mast cell already harbors many IgE’s: there are many subtypes of MastCell–IgE complex. For simplicity, we took “the” MastCell–IgE complex to be a complex of the mast cell with some 100 IgE molecules. This required us to reduce the association rate constant by a factor of 100 to *k*_23_ = 0.1/fM/min. The association rate constant (*k*_22_) of CRA with this “MastCells–IgE complex” should hereby become 100 times the diffusion-limited value of 10^−5^/fM/min for larger molecules. We augmented this by another factor of 100 because of the surface diffusion effects, which we expect to be strong because CRA may already loosely associate with the heavy chains in the IgE receptors. This brought the association rate constant of *R*_22_ to *k*_22_ = 0.1/fM/min. We assumed the breakdown of CRA by MMP-8 (*R*_1_) to be a diffusion-limited bimolecular reaction with a factor of 2 for the attractive effect (55), leading to a rate constant *k*_1_ = 10^−4^/fM/min. For B cells to produce IgE’s they need to be activated by CRA binding to a receptor. We assumed some 15 CRA-receptor molecules to be present on the B cells, leading to a diffusion-limited rate constant for (*R*_9_) of *k*_9_ = 0.001/fM/min. As to the clip-off of CRA from healthy fibroblasts by MMP-7 (*R*_14_), we assumed the encounter of MMP7 with a fibroblast to be diffusion limited at *k*_14_ = 10^−5^/fM/min. This was all done to achieve a system where dying fibroblasts would only exist transiently so as to emit CRA. The killing of healthy fibroblast by TNF-α (*R*_16_) is another bimolecular reaction. Here, we assumed the TNF-α to be able to hit the fibroblast destructively at three sites, making the rate constant equal to *k*_16_ = 0.0005/fM/min. TNF-α was assumed to be released intact after its killing act. Similarly, the antibacterial proteases that are secreted by the mast cells (*R*_25_) were re-released after their killing act (*R*_21_). Here, the biomolecular association rate constant was taken to equal *k*_21_ = 0.005/fM/min, where we assumed >30 sites on each bacterial cell for the protease to attack productively.

### Dissociation Rate Constants *k*_−22_ and *k*_−23_

We expected the CRA and IgE concentrations to be far below 1 fM in the inactive state of the network and then to shoot through the 1 fM level when the systems move into an active state. We therefore set the equilibrium binding dissociation constant in *R*_22_ and *R*_23_ to 1.0 fM by making the corresponding dissociation rate constants *k*_−22_ and *k*_−23_ = 0.1/min.

### Washout Rates: *k*_2_, *k*_4_–*k*_8_, *k*_10_, and *k*_12_

The inflammation was taken to occur in interstitial space. Lymph flow rates led to inverse first order turnover rate constants of 1,200 min in the skin of active rat (58); for skeletal muscle this was 5,000 min. Assuming increased flow at the inflammation site, we took this life time to equal 100 min: all substances other than cells were subject to efflux from the system at the same specific rate of *k* = 1 × 10^−2^/min for the reactions *R*_2_ (*k*_2_), *R*_4_ (*k*_4_), *R*_5_(*k*_5_), *R*_6_ (*k*_6_), *R*_7_(*k*_7_), *R*_8_ (*k*_8_), *R*_10_ (*k*_10_), and *R*_12_ (*k*_12_).

### Secretion Rates and Cell Death Rates: *k*_13_, *k*_14_, *k*_15_, *k*_19_, *k*_20_, *k*_24_, *k*_25_, *k*_26_, and *k*_27_

We assumed the release of MMP-8 by healthy fibroblasts (*R*_20_) to occur in packages of 10,000 enzyme molecules; the reaction stoichiometry of the product MMP-8 was set to 10,000. The same cell was assumed to release MMP-7 (*R*_19_) in packages of 100 molecules at a time. The rates of these two release processes were set to *k*_19_ = 0.001/min for *R*_19_ and *k*_20_ = 0.1/min for *R*_20_. After antigen activation, the MastCells_IgE complex secreted TNF-α (*R*_24_) at a rate constant *k*_24_ = 5/min. The mast cells secreted protease (*R*_25_) at a rate constant of *k*_25_ = 65/min per cell. The healthy and dying fibroblasts secreted CRA (*R*_14_ and *R*_13_) at rate constants of *k*_14_ = 10^−5^/fM/min (see above) and *k*_13_ = 0.001/fM/min, respectively. The dying fibroblasts then disappeared immediately. In a second process, the dying fibroblasts also disappeared at a rate independent of CRA secretion *k*_15_ = 0.2/min (*R*_15_). We assumed that dying bacteria secreted packages of 1,000 CRA molecules at a rate of 10.0/min, *k*_26_ = 10.0/min (*R*_26_). Bacteria died independently of this at a rate constant *k*_27_ = 0.1/min.

### Drug Related Rate Constants *k*_10_, *k*_11_, *k*_−11_, and *k*_12_

In the presence of drug in the system, *R*_10_, *R*_11_, and *R*_12_ were involved accordingly. The two washout rates constants were again taken to equal *k*_10_ = 0.0001/min [washout of drug alone was taken to be 100 times slower than standard washout (see above), thanks to a particular formulation of the drug, see below] and *k*_12_ = 0.0001/min for the same reason. The association rate constant was taken to be limited by diffusion and to equal *k*_11_ = 10.0/nM/min. We set the dissociation rate constant of drug from the IgLC_drug complex to *k*_−11_ = 0.00001/min, setting the half saturating drug concentration to 1.0 fM.

### CRA Influx Rate

Reaction *R*_3_ was set to a constant level. Unless stated otherwise, this level was 0, i.e., *k*_3_ = 0/min.

With the parameter values as stated above, the model was run for various sets of initial conditions and relaxed to the same steady state (with exceptions to be specified below). The magnitudes of all variables were then used as initial conditions for all other calculations, except where we note explicitly that a different starting point was used. For each figure and table, we have a file in the supplementary material that has the definitive COPASI model with the definitive parameter values used such that it can be rerun to reproduce the figure or table. The resulting computed data were analyzed on Graphpad Prism 5.

## Results

### Network Design

Notwithstanding the whole-body nature of innate immunity, we here focus on a local environment in the mammalian body where inflammation may occur, as well as on a set of processes that are relevant for and may suffice to produce inflammation locally. These are described in Figure 1. The innate immune response is held to be activated by CRA (see the dotted line in Figure 1 leading from CRA to the process denoted by *R*_9_), which is inserted into the network as an influx from the outside world (*R*_3_). CRA is secreted by fibroblasts that are activated by TNF-α (see below). In response to CRA, B cells secrete IgE (*R*_9_), which binds to FcεRI receptors on mast cells (*R*_23_). In reaction *R*_22_, we have assumed the FcεRI receptor on the mast cell binding IgE to be activated subsequently by CRA. CRA may also secreted by bacteria (*R*_26_). The mast cell releases mediators such as TNF-α (*R*_24_) and proteases (*R*_25_). When they do not have both IgE and CRA bound, the mast cells do not produce TNF-α; there is no equation describing such a process. Protease production by mast cells is similarly dependent on the latter having both IgE and CRA bound. The proteases may kill bacteria (*R*_21_). Without the proteases the bacteria would proliferate (*R*_17_). TNF-α turns healthy fibroblasts into dying fibroblasts (*R*_16_), which have a greatly enhanced rate of CRA secretion (*R*_13_). Healthy fibroblasts also secrete the proteases MMP-7 (*R*_19_) and MMP-8 (*R*_20_), which catalyze their secretion of CRA (*R*_14_) and the degradation of CRA (*R*_1_), respectively. Fibroblasts are assumed to require free space before they can divide, which is meant to reflect contact inhibition. Free space is a fixed total space minus the volume taken by healthy and dying fibroblasts. It is expressed in unit fibroblast concentration (fM). We assumed that bacteria are not contact-inhibited by fibroblasts.

With respect to the spatial aspects, our model is not defined. It suggests that all processes of Table 1 happen at the site of the inflammation. In reality the B-cells that produce the IgE may be far away from the inflammation site, the CRA released from the fibroblasts reaching the B-cells through the circulation. The IgE produced by those B-cells may then be conveyed back to the inflammation site by circulation. B-cells are known to be present at sites of chronic inflammation and some may already be present at a site of acute inflammation. In such a case, our model could work more locally. A future version of this model should include these spatial aspects as well as the issue that the appearance of CRA may increase the local concentration of IgE producing B-cells.

**Table 1:** Reaction and rate equations for the processes considered in the mathematical model.

*R*_11:_IgE_drug_binding IgE + drug = IgE_drug *v_11_* = *k_11_• IgE • drug − k*_−_*_11_* • *IgE_drug* *k_11_* = *0.01/pM/min; k_−11_* = *0.00001/min*	*R*_12:_IgE_drug_washout IgE_drug + washout -> washout *v_12_* = *k_12_* • *IgE_drug* • *washout* *k_12_* = *0.0001/min*

*R*_13:_CRA_secretion_dyingFibr DyingFibr -> CRA *v_13_* *= k_13_* • *DyingFibr* *k_13_* = *0.001/min*	*R*_14:_CRAClipOffHealthyFibr HealthyFibr + MMP7 -> CRA + MMP7 + HealthyFibr *v_14_* = *k_14_* • *HealthyFibr* • *MMP7* *k_14_* = *0.01/pM/min*

*R*_15:_DyingFibroblast_death DyingFibr -> *v_15_* = *k_15_* • *DyingFibr* *k_15_* = *0.2/min*	*R*_16_:Healthy_to_Dying_fibroblast HealthyFibr + TNF-α -> DyingFibr + TNF-α *v_16_* = *k_16_* • *HealthyFibr* • *TNF-*α *k_16_* = *0.0005/fM/min*

*R*_17_:HealthyBacteriaProduction HealthyBacteria -> 2 * HealthyBacteria *v_17_* = *k_17_* • *HealthyBacteria* *k_17_* = *0.01/min*	*R*_18_:HealthyFibrProduction free_space + HealthyFibr -> 2 * HealthyFibr *v_18_* = *k_18_* • *Free_space* • *HealthyFibr* *k_18_* = *0.485/nM/min*

*R*_19_:MMP7_release_HealthyFibr HealthyFibr -> HealthyFibr + MMP7 *v_19_* = *k_19_* • *HealthyFibr* *k_19_* = *0.001/min*	*R*_20_:MMP7_release_HealthyFibr HealthyFibr -> HealthyFibr + 100 * MMP8 *v_20_* = *k_20_* • *HealthyFibr* *k_20_* = *0.1/min*

*R*_21_:Healthy_to_dying Bacteria HealthyBacteria + Protease -> DyingBacteria *v_21_* = *k_21_* • *HealthyBactmia* • *Protease* *k_21_* = *0.005 fM/min*	*R*_22_:CRA_binding MastCells_IgE + CRA = MastCells_IgE_CRA *v_22_* = *k_22_* • *MastCells_IgE* • *CRA* *−k_22_* • *MastCells_IgE_CRA* *k_22_* = *0.1/fM/min; k_−22_* = *0.1/min*

*R*_23_:IgE_binding IgE + MastCells = MastCells_IgE *v_23_* = *k_23_* • *MastCells_IgE* *−k_23_* • *MastCells_IgE* *k_23_* = *0.1/fM/min; k_−23_* = *0.1/min*	*R*_24_:TNF-α_production MastCells_IgE_CRA -> TNF-α + MastCells_IgE_CRA *v_24_* = *k_24_* • *MastCells_IgE* • *CRA* *k_24_* = 5.0/min

*R*_25_:Protease_production MastCells_IgE_CRA -> Protease + MastCells_IgE_CRA *v_25_* = *k_25_* • *MastCells_IgE_CRA* *k_25_* = *65/min*	*R*_26_:Dying Bacteria secrete antigen DyingBacteria -> 1,000 * CRA *V_26_* = *k_26_* • *Dyingbacteria* *k*_26_ = 10/min

*R*_27_:Dying Bacteria_death DyingBacteria -> *v_27_* = *k_27_* • *Dyingbactetia* *k_27_* = *0.1/min*	

*In the table, each process considered has been given a number, which follows the symbol ‘R’ for reaction (see also Figure 1). A description name is given for each process, followed by both chemical reaction equation, a rate equation, and the parameter values used. Where substances occur on both sides of the arrow in a reaction equation, they are mere (catalytic) influences on the process, without themselves being consumed. This corresponds to the dotted arrows in Figure 1. = refers to a reversible reaction and to a ◊ reaction treated as irreversible*.

Of course, our model is also much simpler than reality with respect to the complexity of the network it takes into account. The network that we have modeled may represent the minimal complexity needed to generate the interesting phenomena that emerge in this paper. In reality there are many, perhaps redundant, pathways of interactions and many additional cell types are involved. Macrophages are not mentioned in our model for instance but are highly relevant in inflammation. We consider that they play roles similar to the role played by the mast cells in or model; in the model the mast cells might be substituted for by macrophages and similar results would come out.

### Network Function

**Figure 2:**
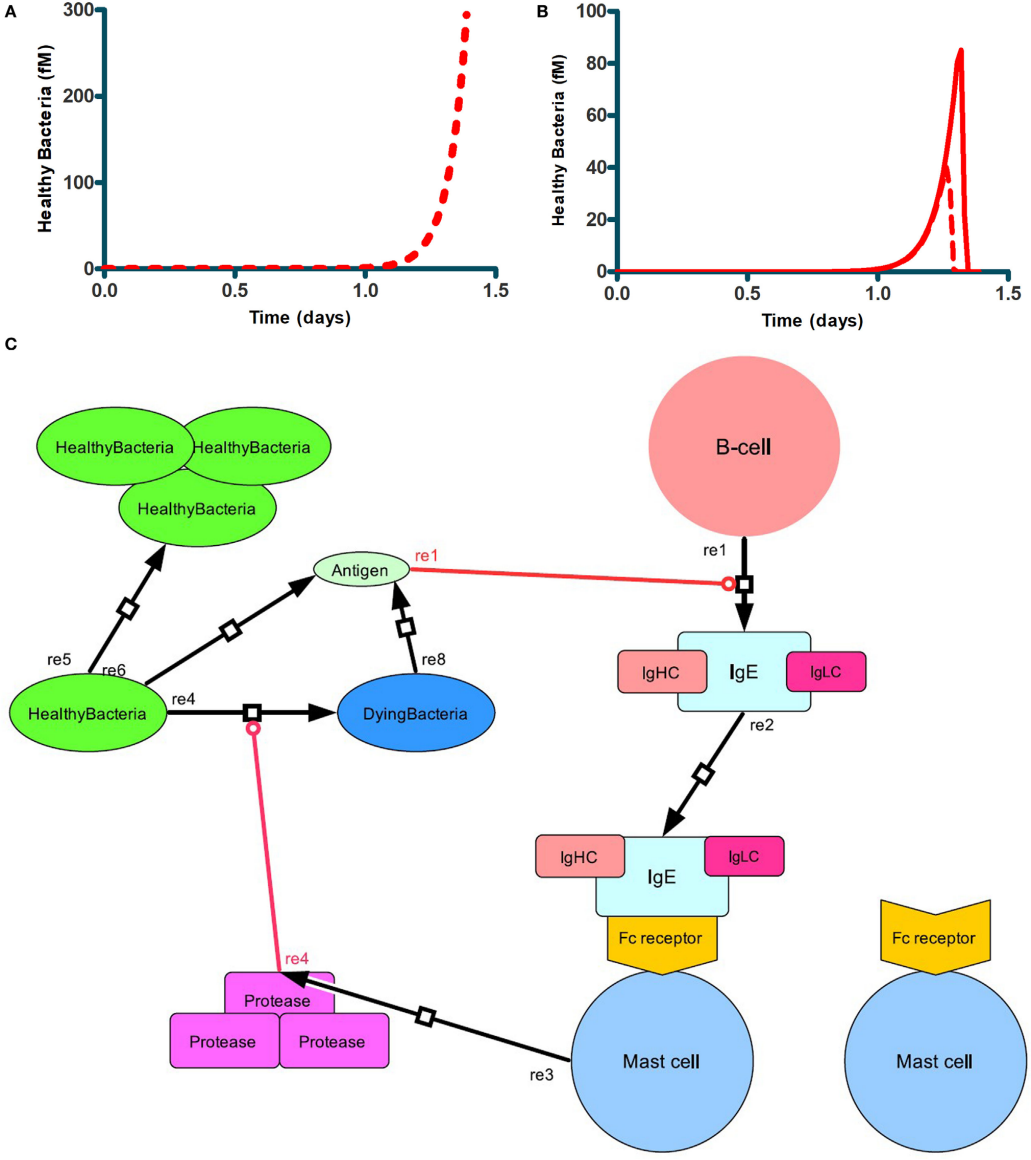
Antibacterial function of mast cells producing proteases. Bacterial growth **(A)** when mast cells produced no protease, i.e., *k*_25_ = 0, or **(B)** did produce protease at a first order rate constant *k*_25_ = 65/min (full red line), or *k*_25_ = 130/min (dashed red line) **(B)**. In both cases, bacteria were seeded into the network at *t* = 0. Mast cell concentration was the standard 0.1 fM. **(C)** Scheme showing the network response to the bacterial challenge.

We first examined whether the network we formulated in Figure 1 would indeed be capable of its perhaps primary task in biology, i.e., to kill invading microorganisms with growth rates outpacing the naïve nonnative immune response. For this we needed to make the network predictive, i.e., to enable all the processes described in Figure 1 to interact in the ways shown in Figure 1, but now in a collective predictive model. For this we formulated a mathematical equation to characterize each process, most often in terms of the rate at which it would proceed (Table 1). Then we formulated balance equations describing the accumulation rate of each substance or cell type as a difference between rates of synthesis and degradation. We then integrated the differential equations produced by combining the rate and the balance equations. This enabled prediction of how the network’s dynamics should be expected to evolve as a function of time and of the values of the various parameters. Figure 2 shows how, for our standard set of parameters, bacteria continued to grow in the control case where the mast cells did not secrete protease (Figure 2A). When mast cells produced the protease continuously, the number of bacteria was reduced relatively suddenly at a later time (Figure 2B) (see also Figure 1). By this, the simulated mast cells succeeded in restraining the microbial population to truly low levels. In our simulation, the time period of virtually disappeared bacteria (approximately 10 days) was followed up by a burst of bacterial growth and a subsequent immune response removing it (results not shown). We suppose that in real life, adaptive immunity would rid the system of such subsequent bursts of microbial growth, but adaptive immunity is not in our model.

### Acute and Chronic Inflammation Produced by the Same Network

**Figure 3:**
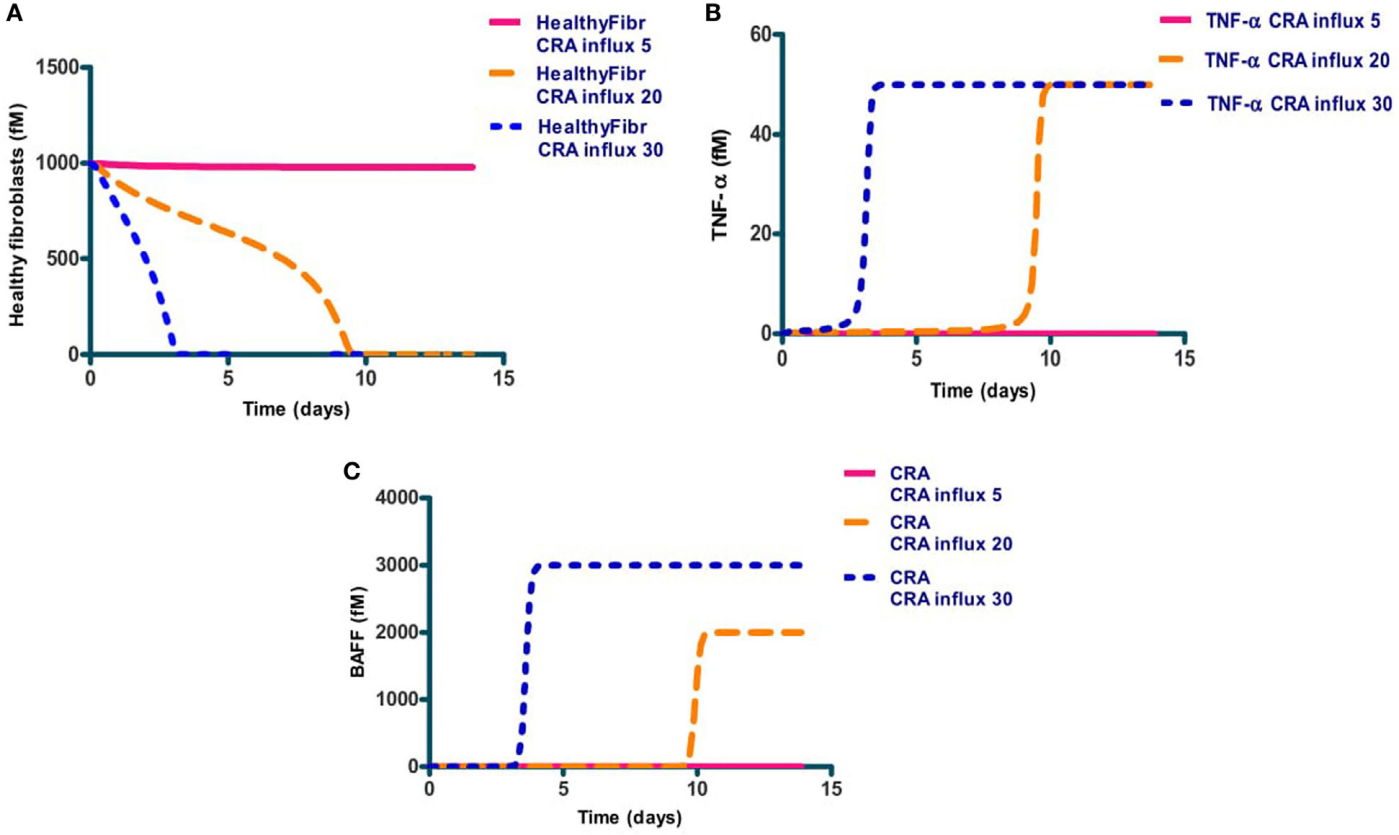
The levels of three network components involved in inflammation, i.e., healthy fibroblasts **(A)**, TNF-α **(B)**, and antigen (CRA) **(C)** as functions of time after increasing (at time 0) the CRA influx level. Full line: no increase in CRA influx, i.e., maintained at 5.0 fM/min. Yellow dashed line: CRA influx increased from 5 to 20 fM/min. The blue dotted line: CRA influx increased from 5 to 30 fM/min.

We next focused on inflammation rather than infection. We studied the response of the network to a modest influx rate of CRA at 5.0 fM/min. We first ran the model until a steady state was obtained and used the resulting magnitudes of all the variables as initial values for the subsequent calculations. The full lines in Figures 3A–C confirm that the steady state obtained for 5.0 fM/min CRA influx was stable: the number of healthy fibroblasts, as well as the TNF-α and CRA levels were independent of time, at high, low, and low levels, respectively, characteristic of a moderately inflamed state. Virtually the same result was obtained for different CRA influx rates between 0 and 12.0 fM/min (not shown). At a CRA influx rate of 20.0 fM/min, however, we observed quite a different behavior (dashed line in Figures 3A–C): already immediately after the increase of the CRA influx, fibroblasts began to die (Figure 3A). This was accompanied by a small but steady increase in TNF-α (Figure 3B) and CRA (Figure 3C) levels with progressing time. Some 8 days after the increase in CRA influx, the rate at which the fibroblasts died began to increase and the TNF-α level increased strongly and suddenly. A day later, the CRA levels also increased suddenly. The next day no live fibroblasts were left (Figure 3A), and the TNF-α and CRA levels had become steady and high. When at time 0, the CRA influx rate was increased by a further 50%, this had little additional effect on the final levels: no live fibroblasts, the same high level of TNF-α, and a steady, even (50%) higher, CRA level. This increase did make a difference to the timing, however, the transition from the low TNF-α, low CRA level to the high levels of these substances, now occurred three times sooner.

**Figure 4:**
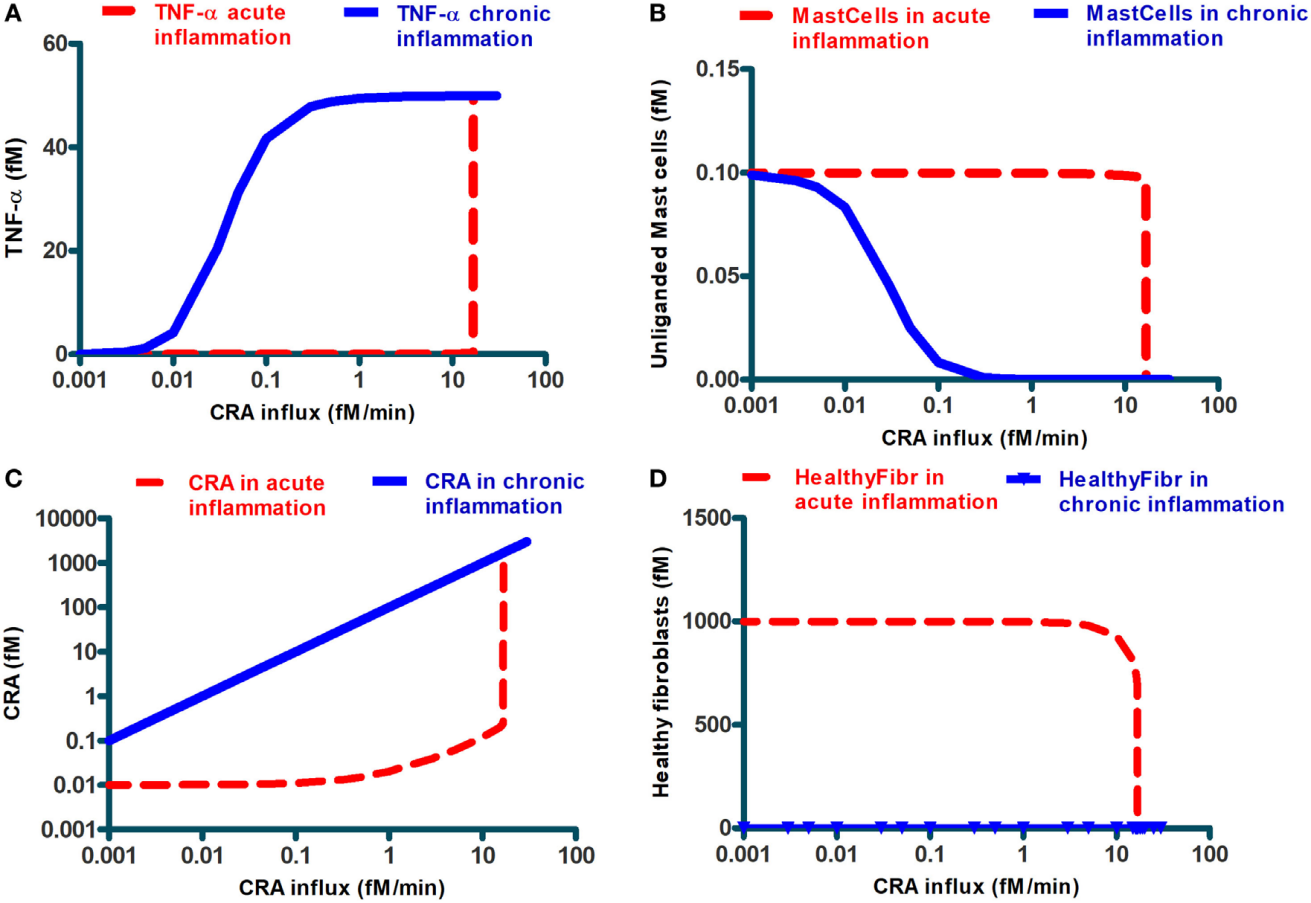
Steady-state levels of various network components at the final steady state achieved when starting from an initial state at very low CRA influx (dashed lines; we call this “acute inflammation”) or starting from an initial state at very high CRA influx (full lines; we call this “chronic inflammation”). The former cases were calculated by starting at a steady state at 0.001 fM/min CRA influx and then increasing that influx to the level indicated on the abscissa. The latter cases were computed by starting at the steady state achieved for 30.0 fM/min CRA influx and by then reducing the CRA influx to the rate indicated on the abscissa (where steady-state concentrations were below 10^–^^9^, which was the resolution of COPASI computation, MMP7 and MMP8, healthy and dying fibroblasts, we reset them to 10^–^^9^ fM). The components are, as indicated, **(A)** TNF-α, **(B)** unliganded mast cells, **(C)** CRA, and **(D)** healthy fibroblasts. The symbols in **(D)** refer to the CRA values for which actual calculations were carried out. In all figures, lines in between were obtained by linear interpolation between calculated points.

Simulation results also suggested a bi-modality in network function. Depending on the intensity of the cytokine challenge, the ultimate steady-state extent of inflammation was either minor or massive and not something in between. The dashed lines in Figure 4 confirm this: they show the final steady-state levels of TNF-α, of mast cells without IgE, of free CRA, as well as of healthy fibroblasts as a function of CRA influx. With all four properties, the same threshold CRA influx rate was evidenced, above which the property flipped to a level that corresponds to strong inflammation, i.e., high TNF-α, low free mast cells, high CRA levels, and few healthy fibroblasts. Only for the CRA levels, the increase with CRA influx rate was, understandably, a bit more gradual.

Up to this point, our calculations started at steady states for low CRA influx rates (5.0 and 0.001 fM/min for Figures 2–4, respectively). The bi-modality we observed above suggested that this might be a case of bi-stability, i.e., a coexistence of two different steady states for the same parameter values and external conditions. Which steady state a bi-stable system settles to, depends on the initial condition. To examine this possibility, we performed a second set of computations, in which we started from the steady state obtained for an influx of 30.0 fM/min of CRA, a state of high inflammatory activity therefore (see Figure 3), and then reduced the CRA load. The solid lines in Figure 4 show the results of these computations: for the entire CRA influx range between 0.05 and 11 fM/min, the steady state achieved in this case was one of strong inflammation, as judged by high levels of TNF-α and CRA and low levels of mast cells without activated IgE, and healthy fibroblasts, whereas for the same conditions the calculations starting at 0.001 fM/min CRA influx all led to minor inflammation. We associated this situation to a dichotomy between acute and chronic inflammation that may occur at apparently the same antigen dosage. Our calculations show that after the network of Figure 1 had been taxed with an overdose of CRA, it tended to remain in the fully inflamed state even if the antigen (CRA) dose was reduced drastically; this is reminiscent of what is observed in chronic inflammation. Figure 4D may be important in particular: in the conditions of chronic inflammation, there are few or healthy fibroblasts left.

### Anti-Inflammatory Peptide

**Figure 5:**
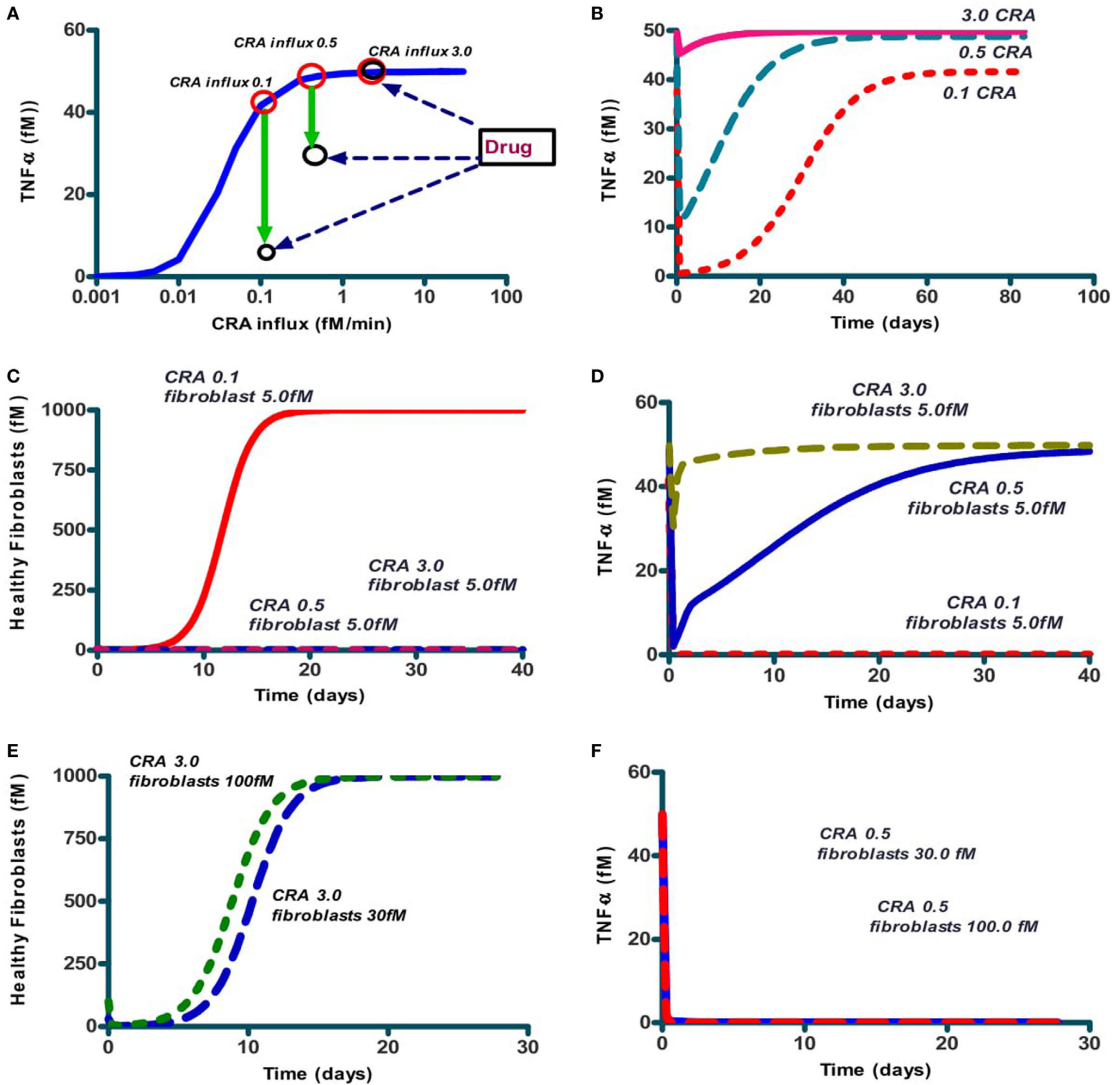
The effect on steady-state TNF-α levels and number of healthy fibroblasts of addition of IgLC-binding peptide to a chronically inflamed network at various sustained rates of CRA influx. **(A)** The full line (blue; also in Figure 4A) represents the steady-state TNF-α levels when no peptide was added; the system had first been brought to the CRA influx steady state of 30 fM/min after which the CRA influx rate was reduced to the number indicated on the abscissa. This produced the chronic inflammation state even for the lower CRA influx rates. The red circles indicate these original chronic inflammation states at CRA influx rates of 0.1, 0.5, and 3.0 fM/min. The downward green arrows to the black circles show the effect after 15 simulated days, of then adding 1 nM of IgLC-binding peptide at sustained CRA influx rates of 0.1, 0.5, or 3 fM/min. **(B)** The time dependence of TNF-α after peptide addition to any of these three chronic inflammation states, i.e., at CRA influxes of 0.1, 0.5, and 3 fM/min. **(C)** The effect on fibroblast levels of 5.0 fM fibroblast addition to the system at steady state simultaneously with peptide addition, of the 0.1 (full red line), 0.5 (dashed blue line), and 3.0 (dashed red line) fM CRA/min chronic states as in **(A)**. **(D)** The effect on TNF-α levels of 5 fM fibroblasts added to the system at steady state simultaneously with drug treatment of the CRA influx of 0.1 (dashed red line), 0.5 (blue full line), and 3.0 (dashed green line). **(E)** Fibroblast concentration as function of time after injecting 6 times (dashed line) or 20 times (dotted line) more healthy fibroblasts to the chronic 3.0 fM CRA/min state. **(F)** The effect on TNF-α levels of giving 30 or 100 fM fibroblasts to the system at steady state simultaneously with peptide treatment at CRA influx rates of 0.5 (red dotted line), 3.0 fM/min (blue full line, coinciding with axes).

A possible explanation of the bi-stability might be the positive feedback loop of CRA upon itself, through activation of mast cells by CRA binding to IgE presented by those cells, TNF-α elevation and CRA production by dying fibroblasts (Figure 1B). Such loops can produce thresholds below which a system tends to one state and above which it prefers another. We wondered whether interference with that feedback loop could do away with chronic inflammation, and if so, whether that interference would have to have special qualifications. The solid line in Figure 5A repeats that of Figure 4A, i.e., it shows the TNF-α level after reducing CRA influx rates back from 30 to the value indicated on the abscissa. The red circle on the right in Figure 5A gives the TNF-α level attained (after 15 simulated days) when, to a chronic inflammation system with a continuous CRA influx of 3.0 fM/min (as indicated on the abscissa), a single shot of IgLC-binding peptide was given (see Figure 1). Figure 5A shows that perhaps unexpectedly, the peptide drug was ineffective even though the CRA influx was well below the CRA influx threshold of 12 fM/min of Figure 4A. In the chronic inflammation state with a CRA influx rate of 0.1 fM/min, however, the system did respond to the peptide-based drug, and it did so by what appeared, after 15 simulated days, to be a switch to the low-inflammation state (the lower black circle on the left in Figure 5A).

We then wondered whether this flipping back by the drug to the acute inflammation state would be definitive, as expected from a bi-stable steady-state situation. We therefore followed the TNF-α level for a long time (Figure 5B). We found that the flip was not definitive: in some 30 days, the system drifted back to the state of high inflammation. This was a bit odd: we seemed not to have a bi-stable state of high inflammation and low inflammation: after the history of high inflammation the low-inflammation state was no longer stable, but transient, though apparently stable for a couple of days. Apparently, the transition from low to high inflammation at high CRA influx rates had been irreversible.

We next searched for the factor that might be the culprit of the irreversibility and turned to the fibroblasts. These had actually gone extinct in the high inflammation state. In our model, new fibroblasts could only appear through division of existing fibroblasts, so that the killing of all fibroblasts that the model produced should be irreversible. In reality, however, fibroblasts from neighboring tissues might come to the rescue by invading. In order to test this idea, we then added a low concentration of fibroblasts together with the peptide to the model. Indeed, for the lower CRA influx rate of 0.1 fM/min, addition of very few healthy fibroblasts (5 fM) already triggered their regrowth to full confluence, as shown in Figure 5C. Conversely, the levels of healthy fibroblasts went back to 0 at sustained CRA influx rates of 0.5 and 3 fM/min, as shown in Figure 5C as well. Figure 5D shows that with the high CRA influx rates also TNF-α came back to its maximum level. The addition of more healthy fibroblasts (30 and 100 fM) resulted also in the latter cases of 0.5 and 3 CRA influx in regrowth of the fibroblasts (Figure 5E) and reduction of TNF-α (Figure 5F).

**Figure 6:**
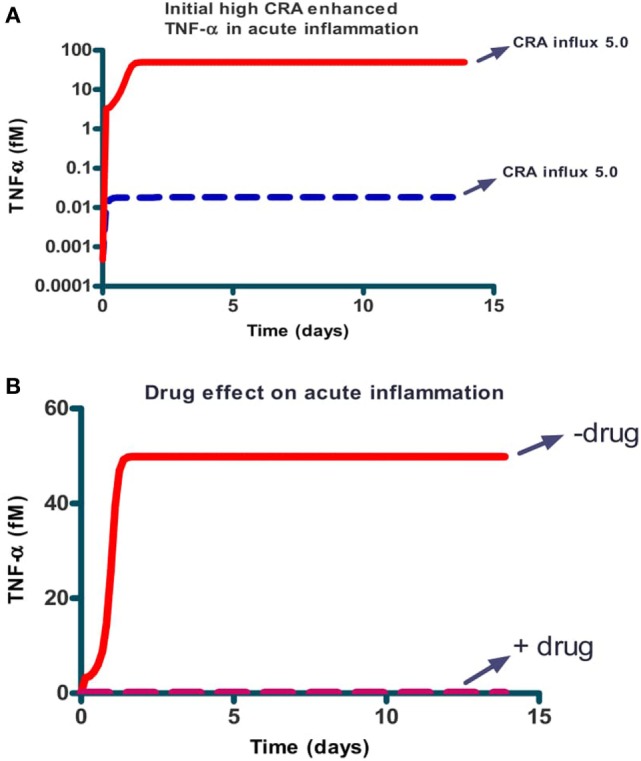
Prevention: the effect of a single dose of CRA on steady-state TNF-α level for the case of acute inflammation in the absence **(A)** and presence [**(B)** dashed line] of a single dose of IgLC-binding peptide. **(A)** Simulations started at an acute inflammation steady state with 5.0 fM/min CRA influx (as in Figure 4A) and a bolus of CRA was added at this time 0. The amounts added were 0.01 fM and 10 µM for the dashed blue and full red lines respectively. **(B)** 10 µM of CRA was added at time 0 together with (dashed red line) or without (full red line) 1.0 nM of peptide. CRA influx was maintained at 5.0 fM/min in all cases.

In the situation above, peptide addition was able to switch the network back from its chronic inflammation state to a virtually uninflamed state, either transiently (when no fibroblasts were reseeded) or persistently (when they were). We also wished to examine whether peptide addition could be used for prevention, i.e., prevent the network from turning into the chronic inflammation state altogether. We therefore set the CRA influx rate at 5 fM/min and started the computation from the hardly inflamed state. We added a bolus of 10 µM CRA and found (Figure 6A) that for initial amounts of CRA below 1 µM the system returned to the hardly inflamed steady state characterized by low TNF-α (dashed blue line). For initial levels of CRA exceeding this 1 µM, the system still became chronically inflamed (the full red line). The Figure 6B shows that the IgLC-neutralizing peptide was able to prevent the switch to full-blown inflammation induced by 10 µM CRA. Specifically, the drug prevented the dying of healthy fibroblasts (not shown) and brought the TNF-α level back to 0.01 or virtually 0.

### Inter-Individual Differences

**Figure 7:**
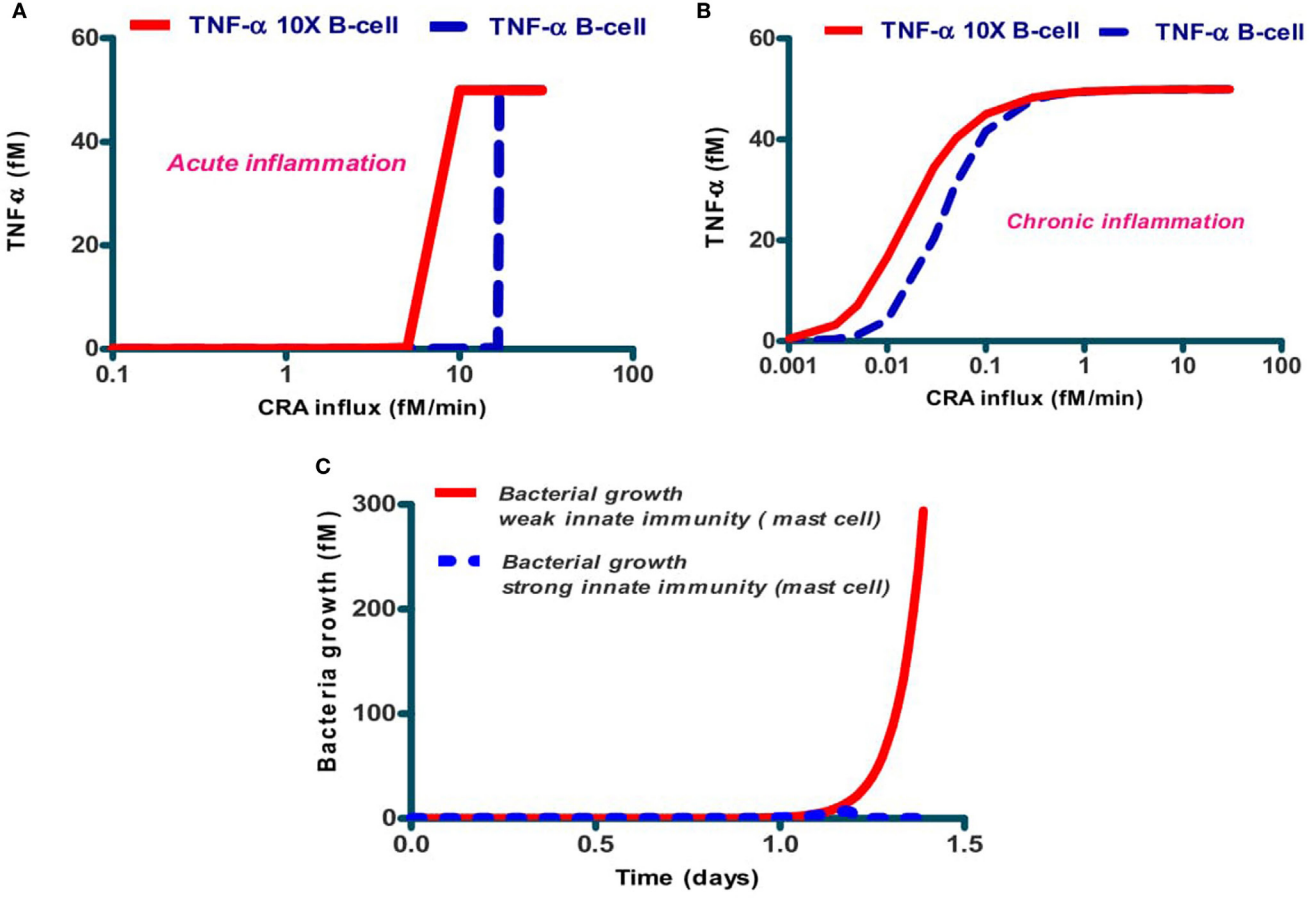
Dependence of acute **(A)** and chronic **(B)** inflammation on the rate of CRA influx and in bacterial infection **(C)** depends on the individual's B-cell level. The dashed lines in **(A,B)** refer to the standard case, while the full lines refer to the same calculations but for five times increased B-cell levels. **(C)** Dependence of host response *in silico* on the level of mast cells. The full red line refers to the standard case with 0.1 fM of mast cells, and the dashed blue line (almost coinciding with the abscissa) to the case with 1 fM of mast cells. Protease secretion rate constant for mast cells was *k*_25_ = 65/min and B-cell concentration was 1 fM in both cases.

The activities of the various components of the native immune network may differ between human individuals. We examined whether the dichotomy between chronic and acute inflammation predicted by Figure 4 for the network of Figure 1, would depend on B-cell activity, which does differ between individual humans due to differences in genome or because of different pre-exposure to CRA like antigens. Figure 7A shows that for the case of acute inflammation (i.e., a calculation starting at low CRA influx rates), the TNF-α steady state level attained is already high at much lower CRA influx levels when the individual was modeled to have 10 times more B cells. A similar effect was computed for the dependence of TNF-α on CRA influx rate in the chronic inflammation case (Figure 7B). In addition, we simulated for the bacterial growth a time course in two cases to test whether such inter-individual differences should also be expected to affect the strength of innate immunity *per se* (Figure 7C).

## Discussion

Tumor necrosis factor-α, fibroblast killing, and mast cell activation are all associated with inflammation, but inflammation itself can assume dichotomous forms. The one is witnessed by a moderate increase in TNF-α and other cell lytic factors, just enough to kill a population of invading microorganisms or to rid the body of a damaged piece of tissue. It is also transient and disappearing when the microorganisms or damaged tissue have been removed. This is the acute type of inflammation. The other type is sustained, persisting long after the antigenic challenge has disappeared, and continuing to remove tissue, including healthy tissue of the individual itself. This chronic inflammation is associated with diseases such as rheumatoid arthritis (RA). It may depend on cross-reactivity between exogenous and endogenous antigens, taking the place of CRA in our Figure 1. The distinction between acute and chronic inflammation is a bit unclear when it is accepted that the intensity of the acute response increases with the intensity of the antigenic challenge. What then is the difference between a strong acute inflammation caused by a high amount of CRA and a perhaps weaker chronic inflammation in the presence of a lower amount of antigen? Indeed, why is it that when exposed to a given antigenic dose the one person exhibits minor but useful inflammation whereas another person is subject to strong and pathological inflammation?

To try to understand these paradoxical phenomena in the innate immune response *vis-à-vis* the confusingly high number of factors involved, we put some of the important factors together into a much simplified map of the system (Figure 1) and then made this map predictive by integrating corresponding mathematical equations. The calculations were indeed predictive of function: they predicted (i) that the network would act successfully against bacterial infections, (ii) that CRA would produce inflammation also in the absence of bacteria, (iii) that there are two types of inflammation, and (iv) that a peptide-binding IgLC could redress inflammation from chronic to acute or could prevent chronic inflammation from occurring when the network was challenged by a high dose of CRA.

All these predictions are paralleled by validations in the experimental literature. For (i)–(iii), these are common knowledge; we now have a model-supported understanding of these features of innate immunity. Issue (iv) is a more specific test, where indeed one of us demonstrated that anti-IgLC peptide injected i.p. into mice reduced ear swelling caused by previous injection of antigen (33).

In a previous note (59), one of us referred to this finding as well as to findings by Dispenzieri et al. (60), as evidence of a much greater role of IgLC in innate immunity and in fact non-specific human mortality than previously assumed. With the present paper, we have now underpinned substantiated this proposal: this may indeed a role for IgLC as component of IgE that follows from the integration of the relevant data through a systems biology model.

This validation of the four main model predictions requires qualifications. The first is that the network we made predictive (i.e., Figure 1) is far from completely representing all the factors involved in innate immunity and from presenting all interactions in the network properly. One issue is that we held CRA as essentially covariant with multiple antigens. A second is that the parameter values inserted in the model may not have been the precise actual parameter values, because most of the latter are insufficiently known. Consequently, the present study should be seen as a mere demonstration of what networks of innate immunity might be capable of. It also offers a first innate-immunity network that exhibits the essential functional properties mentioned above, amenable to *in silico* prediction of actually observed behavior. A third issue is that the model is not spatially defined, assuming CRA to be in contact with B-cells that might be in bone marrow (see the model description above). And, some relevant cell types are not mentioned, such as the macrophages. They may play roles similar to the mast cells, which would be simulated by the model, but then again, they will add additional aspects that we did not model here.

### The Parameter Values

Having written the above *caveat*s, we would add that the parameter values used are of interest, because they were chosen and argued to be as realistic as possible with current knowledge. Cell cycle times were realistic, association rate constants were taken to reflect diffusion limitation but with corrections for multiple molecular targets on the same target cell, surface diffusion, and attractive forces. Protease secretion by mast cells was modeled to occur in batches. Our model hereby differs from other models in mathematical biology in that it is based on realistic estimates of the parameter values rather than on values obtained by fitting. The observation that the model reproduces so many features of immunology, suggests that we are not that far away from the actual parameter values. As such, the network and the parameter values, we used may serve future studies where parameter values are determined experimentally. In this sense, the model presented here may serve as a facility: for each figure and table, we have added a file in the supplementary material that has the definitive COPASI model in which the parameter values can be adjusted. Models are also in JWS-Online (jjj.bio.vu.nl) to new experimental data, such that implications of the new data can be computed.

### CRA Influxes and Acute Inflammation

Our computational study showed that the network flipped to a state of very high inflammation at high challenges with antigen influx, with very high levels of TNF-α and extensive cell death as a result. This complex association is perhaps best illustrated by Figure 4C, where we see the predicted level of CRA (antigen) as a function of CRA influx. The correlation between disease and CRA appears to run in two directions: first, increasing influx of CRA leads to little inflammation until the CRA influx exceeds a threshold, and then (see the dashed line in Figure 4A) only with a minor further increase in CRA influx, inflammation becomes much stronger: high CRA influx associates with highly intensive inflammation.

### Acute Versus Chronic Inflammation

Acute inflammation can turn into chronic inflammation (61, 62). If an acute inflammation has not resolved itself in days, it can thereby be considered chronic. This phenomenon is predicted by our computations of the implications of the network of Figure 1 with realistic parameter values: as pointed out in Figures 3 and 4, continued acute inflammation caused by a continued influx of CRA at an intensity level above a certain threshold, is predicted to lead to strong inflammation with much elevated TNF-α. Once steady state has been achieved for this strong and acute inflammation, return of the CRA influx to very low levels, fails to lead to a reduction in TNF-α levels (see Figure 4A, the full line): substantial exposure of the network to external CRA above a certain threshold intensity does not only produce a strong acute inflammation but also turns the network into a chronic inflammation state. Consequently, for the same antigenic challenge the network can turn to a high or a low inflammation state, depending on its history. This suggests a mechanistic basis of the difference between acute and chronic inflammation in the quasi bi-stability of the network of Figure 1.

### Targeting Inflammation

*Vis-à-vis* medication, innate immunity and its active components are frequent targets (63, 64). The mast cell contributes many different mediators, including tryptase, VEGF, and TNF-α. These are contributors to tissue inflammation (65–69). We here examined if our predictive network facility could serve in pre-validating *in silico*, therapies one might propose for chronic inflammation and whether an *in silico* study might provide early warnings of complications of such a therapies. We focused on a peptide binding to the IgLC part of IgE inhibiting indirectly the activation of mast cells by CRA. Sure enough, we found that such a peptide could switch the network back from a chronic to an acute inflammatory state. However, the effect of the peptide was transient only, thereby requiring multiple dosing of the peptide at subsequent points in time, or a very low drug washout rate for that effect to persist acceptably long (results not shown).

In this paper, we assumed that the peptide drug would interact with the IgLC that is part of IgE. In a different incarnation of the model, the role of IgE would be played by immunoglobulin heavy chain devoid of light chain, which would then bind to the mast cells, after which there could be piggy-back binding of the FLC molecules. B-cells would then not only secrete FLCs but also HCs, which should then be able to bind to IgE receptors on the mast cells (67). Both this mechanism and the mechanism we used in the present paper, should lead to similar kinetic models and hence to the same results as shown in this paper.

At a lower CRA influx load the peptide had a longer lasting but still no permanent effect. This was because in our simulations the fibroblasts had gone extinct. Consequently, the system could not return to the uninflamed or acutely inflamed steady state. Why would the fibroblasts matter, however? The fibroblasts themselves are not directly involved in stopping inflammation. The solution to this riddle was (not shown) that the fibroblasts also produce MMP-8 which is a protease that removes CRA and thereby defuses the inflammation. Our simulations in which we did not only add drug but also fibroblasts to the system, confirmed this and suggest that the fibroblasts play an important role in keeping inflammation low by scavenging, through the proteases they secrete, the inflammatory antigens.

Economically and in view of a possible immune response against the peptide, therapy with a single dose of peptide could be preferable. Perhaps such a therapy could just consist of adding peptide and rely on fibroblasts growing back in from the surroundings of the inflamed tissue. But again perhaps, it should be advisable to try and activate this repopulation by fibroblasts. A further analysis using patient data and our model might be able to predict whether the CRA levels in a particular patient would indeed advocate a one-time high-dose treatment with peptide rather than a continuous treatment at a lower dose, or treatment combined with repopulating with fibroblasts.

For the absence of fibroblast implantation, the network predicts the anti-inflammatory effect of the peptide to last only for a month or so. This finding may be important, because it could, wrongly, suggest that drug resistance has arisen. Here the mechanism is that the drug has not *irreversibly* flipped the network back from its chronic to its acute inflammation state. We also examined a different mode of treatment, i.e., one that might prevent acute inflammation turning into chronic inflammation. Here peptide added when the acute high antigen dose arose, was predicted to be effective. This was not what we found for all levels of CRA, however: In all these cases, there is a threshold dependence on dose of both CRA and peptide, as well as a complex time-dependence. Deciding on the precise dosing and dynamics of the therapy and on its limitations, might benefit from having a model such as the one developed here, available.

### Individualized Disease

B cells play a vital role in both acute and chronic inflammation (70–72). We found that at a 10-fold increase in B-cell concentration decreased the threshold CRA influx rate above which inflammation intensity switched from low to high: the window of CRA influx rates where the network was bi-stable between chronic and acute inflammation was then shifted to lower CRA influx rates. This illustrates that the phenomena of acute and chronic inflammation and the effectiveness of therapies may differ significantly but also predictably, between individual patients.

### Complex Regulation

The network in Figure 1 contains a positive feedback loop, which involves CRA activation of mast cells into the secretion of TNF-α, and stimulation by TNF-α of CRA secretion by fibroblasts. A complicating matter is that the mediators of CRA secretion are themselves compromised by TNF-α. We have here shown a way to deal with the complexity of this type of regulation, i.e., by making predictive models and by simulation *in silico*. This has enabled us to show that the complex regulation also results in complex, yet functional properties. One such property of the network is that there is an appreciable range of CRA levels for which the inflammation remains acute. Only at the highest CRA levels, beyond an “On” threshold, a switch occurs to highly intense inflammation, which may then lead to chronic inflammation when maintained sufficiently long. Conversely, we also found that there is a switch back from the chronic inflammation state to the state of acute inflammation, but that this switch requires (i) reduction of the CRA load (as modeled by CRA influx) to an intensity below an “Off” threshold that is far below the “On” threshold and (ii) fibroblast repopulation. This “Off” threshold is a bit more gradual. This information may re-motivate therapies of antigen removal that failed previously: because of the dual threshold effect, a slightly more intensive antigen reduction might prove more effective than a longer lasting therapy with a lower concentration of peptide. Indeed a more sophisticated approach to drug administration may be enabled by predictive modeling and this might improve the therapy of chronic inflammation. Monitoring of more of the properties involved, such as those of CRA and TNF-α, would further empower such an approach.

Our simulations also suggest that pathology and optimal therapy should be expected to differ between individual patients (our example was that of differences in B-cell activity). Use of improved versions of our model and of biomarkers interpreted through these models may aid diversified and personalized drug development. Similar modeling may help interpret experiments inquiring whether a similar positive feedback loop accomplishes a specific activation of other innate immune cells such as neutrophils, macrophages, and basophils and whether this may also occur in the vicinity of a tumor (73, 74).

## Author Contributions

AA: developed the model together with HW, carried out the simulations, and wrote the paper together with HW. MB: checked the model. FR: advised on the immunology with respect to the peptide drug. NS: contributed to the design of the network. HW: developed the model together with AA, helped write the paper, and supervised the whole work.

## Conflict of Interest Statement

The authors declare that the research was conducted in absence of any commercial or financial relationships that could be construed as a potential conflict of interest.
