# Supplementary material for: Predictable Irreversible Switching Between Acute and Chronic Inflammation
Source: Front Immunol. 2018 Aug 7;9:1596. doi: 10.3389/fimmu.2018.01596 (PMC6090016; doi:10.3389/fimmu.2018.01596)
Supplement: Supplementary file 1 [file data_sheet_1.pdf]

Supplementary Material

Predictable Irreversible Switching between Acute and Chronic Inflammation

Abulikemu Abudukelimu, Matteo Barberis, Frank Redegeld, Nilgun Sahin, Hans V. Westerhoff*

*** Correspondence:** Prof. dr. Hans V. Westerhoff: Hans.Westerhoff@manchester.ac.uk or H.V. Westerhoff@UvA.NL

Supplementary Data:

1. **Table 1: Key molecules in the network**
2. **Correspondence between names**
3. **Initial conditions for standard model**
4. **The models**

1. **Supplementary Table 1- key molecules in the network**

Abbreviation	Description	References
**CRA**	Cross-reacting antigen	Ng LG *et al (2)*; Krumbholz *et al (3)*; Zouggari Y *et al (4)*
**FLC**	Free light chain	Redegeld *et al (5)*; Rijnierse *et al (6)*; Maurer *et al (7)*
**MMP-7**	Metalloproteinase-7	Ii *et al (8)*; Slattery *et al (9)*; Zeng *et al* (10)
**MMP-8**	Metalloproteinase-8	Vayrynen *et al (11)*; Thirkettle *et al (12);* Quintero *et al (13)*
**TNF-α**	Tumor necrosis factor-alpha	Bradley *et al (14)*; Chastre *et al (15)*; Jo *et al (16)*

1. Choi, S., and SpringerLink (Online service). Systems Biology for Signaling Networks. In *Systems Biology*,. XVI, 908p. 326 illus., 910 illus. in color.

2. Ng, L. G., A. P. Sutherland, R. Newton, F. Qian, T. G. Cachero, M. L. Scott, J. S. Thompson, J. Wheway, T. Chtanova, J. Groom, I. J. Sutton, C. Xin, S. G. Tangye, S. L. Kalled, F. Mackay, and C. R. Mackay. 2004. B cell-activating factor belonging to the TNF family (BAFF)-R is the principal BAFF receptor facilitating BAFF costimulation of circulating T and B cells. *J Immunol* **173**: 807-817.

3. Krumbholz, M., and E. Meinl. 2014. B cells in MS and NMO: pathogenesis and therapy. *Semin Immunopathol* **36**: 339-350.

4. Zouggari, Y., H. Ait-Oufella, P. Bonnin, T. Simon, A. P. Sage, C. Guerin, J. Vilar, G. Caligiuri, D. Tsiantoulas, L. Laurans, E. Dumeau, S. Kotti, P. Bruneval, I. F. Charo, C. J. Binder, N. Danchin, A. Tedgui, T. F. Tedder, J. S. Silvestre, and Z. Mallat. 2013. B lymphocytes trigger monocyte mobilization and impair heart function after acute myocardial infarction. *Nat Med* **19**: 1273-1280.

5. Redegeld, F. A., M. W. van der Heijden, M. Kool, B. M. Heijdra, J. Garssen, A. D. Kraneveld, H. Van Loveren, P. Roholl, T. Saito, J. S. Verbeek, J. Claassens, A. S. Koster, and

F. P. Nijkamp. 2002. Immunoglobulin-free light chains elicit immediate hypersensitivity-like responses. *Nat Med* **8**: 694-701.

6. Rijnierse, A., F. A. Redegeld, B. R. Blokhuis, M. W. Van der Heijden, A. A. Te Velde, I. Pronk, D. W. Hommes, F. P. Nijkamp, A. S. Koster, and A. D. Kraneveld. 2010. Ig-free light chains play a crucial role in murine mast cell-dependent colitis and are associated with human inflammatory bowel diseases. *J Immunol* **185**: 653-659.

7. Maurer, M. J., I. N. Micallef, J. R. Cerhan, J. A. Katzmann, B. K. Link, J. P. Colgan, T. M. Habermann, D. J. Inwards, S. N. Markovic, S. M. Ansell, L. F. Porrata, P. B. Johnston, G. S. Nowakowski, C. A. Thompson, M. Gupta, S. I. Syrbu, P. J. Kurtin, W. R. Macon, D. A. Nikcevich, and T. E. Witzig. 2011. Elevated serum free light chains are associated with event-free and overall survival in two independent cohorts of patients with diffuse large B-cell lymphoma. *J Clin Oncol* **29**: 1620-1626.

8. Ii, M., H. Yamamoto, Y. Adachi, Y. Maruyama, and Y. Shinomura. 2006. Role of matrix metalloproteinase-7 (matrilysin) in human cancer invasion, apoptosis, growth, and angiogenesis. *Exp Biol Med (Maywood)* **231**: 20-27.

9. Slattery, M. L., E. John, G. Torres-Mejia, M. Stern, A. Lundgreen, L. Hines, A. Giuliano, K. Baumgartner, J. Herrick, and R. K. Wolff. 2013. Matrix metalloproteinase genes are associated with breast cancer risk and survival: the Breast Cancer Health Disparities Study. *PLoS One* **8**: e63165.

10. Zeng, Z. S., W. P. Shu, A. M. Cohen, and J. G. Guillem. 2002. Matrix metalloproteinase-7 expression in colorectal cancer liver metastases: evidence for involvement of MMP-7 activation in human cancer metastases. *Clin Cancer Res* **8**: 144-148.

11. Vayrynen, J. P., J. Vornanen, T. Tervahartiala, T. Sorsa, R. Bloigu, T. Salo, A. Tuomisto, and M. J. Makinen. 2012. Serum MMP-8 levels increase in colorectal cancer and correlate with disease course and inflammatory properties of primary tumors. *Int J Cancer* **131**: E463-474.

12. Thirkettle, S., J. Decock, H. Arnold, C. J. Pennington, D. M. Jaworski, and D. R. Edwards. 2013. Matrix metalloproteinase 8 (collagenase 2) induces the expression of interleukins 6 and 8 in breast cancer cells. *J Biol Chem* **288**: 16282-16294.

13. Quintero, P. A., M. D. Knolle, L. F. Cala, Y. Zhuang, and C. A. Owen. 2010. Matrix metalloproteinase-8 inactivates macrophage inflammatory protein-1 alpha to reduce acute lung inflammation and injury in mice. *J Immunol* **184**: 1575-1588.

14. Bradley, J. R. 2008. TNF-mediated inflammatory disease. *J Pathol* **214**: 149-160.

15. Chastre, A., M. Belanger, E. Beauchesne, B. N. Nguyen, P. Desjardins, and R. F. Butterworth. 2012. Inflammatory cascades driven by tumor necrosis factor-alpha play a major role in the progression of acute liver failure and its neurological complications. *PLoS One* **7**: e49670.

16. Jo, M., T. H. Kim, D. W. Seol, J. E. Esplen, K. Dorko, T. R. Billiar, and S. C. Strom. 2000. Apoptosis induced in normal human hepatocytes by tumor necrosis factor-related apoptosis-inducing ligand. *Nat Med* **6**: 564-567.

2. Supplementary Table of name correspondences

The models have been developed for two instantiations of substances. Correspondence between the names are:
BAFF=CRA
FLC = IgE
FLC_Drug = IgE_drug
MastCells_FLC_CRA = MastCells_IgE_CRA

3. Initial conditions for standard model

The model that led to the dashed line in Fig. 4A had the initial conditions for the variables as given in the following Table. Initial conditions for the other Figures were the same except as indicated in legend or text. An example of a different initial conditions are the calculations that led to the full blue line in Fig. 4A, where the steady state computed for the standard state at 30 fM/min CRA influx was used as initial condition. All precise initial conditions can be found in the Copasi models.

Name	Compartment	Type	Initial Concentration (fmol/l)	Concentration (fmol/l)
CRA	compartment	reactions	0.00999412	nan
MMP8	compartment	reactions	999509	nan
washout	compartment	reactions	1	nan
IgE	compartment	reactions	0.000999412	nan
MMP7	compartment	reactions	99.951	nan
Protease	compartment	reactions	1	nan
Bcells	compartment	fixed	1	1
drug	compartment	fixed	0	0
IgE_drug	compartment	fixed	0	0
DyingFibr	compartment	reactions	0	nan
HealthyFibr	compartment	reactions	999.509	nan
TNFalpha	compartment	reactions	0.000475162	nan
HealthyBacteria	compartment	fixed	0	0
free_space	compartment	assignment	0.491	nan
DyingBacteria	compartment	fixed	0	0
MastCells_IgE	compartment	reactions	9.98404e-05	nan
MastCells_IgE_CRA	compartment	reactions	9.97816e-07	nan
MastCells	compartment	reactions	0.0998992	nan
Total space	compartment	fixed	1000	1000

4. The models

For all Figures the Copasi models have been uploaded as separate supplementary material in a zipfile. Some of the models may use different names (instantiations) for the same mathematical instantiations (see ‘name correspondences above)’.
